# Optimizing emergency nursing protocols to enhance outcomes in patients with acute myocardial infarction: A retrospective study

**DOI:** 10.1097/MD.0000000000041412

**Published:** 2025-06-06

**Authors:** Fang Yu, Xueqin Yuan, Shouzhi Fu

**Affiliations:** a Department of ICU/Emergency Wuhan Third Hospital, Tongren Hospital of WuHan University, Wuhan, China.

**Keywords:** AMI, emergency nursing, myocardial enzyme

## Abstract

This study evaluates the impact of an optimized emergency nursing process on treatment outcomes, prognosis, patient satisfaction and analyzes the relationship between shortened rescue time and myocardial enzyme levels in patients with acute myocardial infarction (AMI). This retrospective study included 201 patients with AMI admitted to our hospital’s emergency department between June 2022 and February 2024. Patients were divided into an observation group (n = 93), which received optimized emergency nursing care, and a control group (n = 108), which received standard care. The optimized nursing process encompassed prehospital emergency intervention, rapid transportation, in-hospital treatment, continuous monitoring, and psychological support. Data collected included rescue times, treatment effectiveness, anxiety and depression scores, patient satisfaction, and myocardial enzyme levels. The correlation between rescue time and myocardial enzyme levels was analyzed. Compared with the control group, the observation group demonstrated significantly shorter rescue times, including triage assessment and emergency electrocardiogram examination (*P* < .05). Clinical outcomes such as ST-segment resolution, chest pain relief, and myocardial enzyme normalization were notably better in the observation group (*P* < .05). A significant negative correlation was found between rescue time and myocardial enzyme levels in both groups, with correlation coefficients of −0.41 in the observation group and −0.29 in the control group. This indicates that shorter rescue times are associated with faster myocardial enzyme recovery. In addition, the observation group reported significantly lower anxiety and depression levels, higher hope scores, and greater patient satisfaction (*P* < .05). Optimizing the emergency nursing process significantly improves treatment outcomes and prognosis in patients with AMI by reducing rescue times, enhancing myocardial enzyme recovery, and improving psychological well-being and patient satisfaction. The significant negative correlation between rescue time and myocardial enzyme levels underscores the importance of rapid emergency care. These findings support the implementation of this optimized approach in clinical settings to improve AMI management and patient outcomes.

## 1. Introduction

Every year, over 1 million people die from heart attacks and related complications, with >600,000 new cases of acute myocardial infarction (AMI) diagnosed annually. Patients with AMI often present with severe symptoms such as chest pain, circulatory dysfunction, and heart failure, all of which can rapidly escalate to life-threatening complications, including sudden cardiac death. The primary treatment for AMI is reperfusion therapy, aimed at restoring blood flow to ischemic cardiac tissue.^[1–3]^ The effective “golden window” for treatment is generally considered to be within 2 hours of symptom onset. However, many patients miss this critical window due to delays in transportation to the hospital and prolonged waiting times for treatment, leading to poor prognoses.^[4]^

As a result, there has been a concerted effort to optimize emergency care protocols to reduce delays, expedite treatment, and improve survival rates for patients with AMI. Emergency care for AMI requires a comprehensive approach, including prehospital care, rapid transportation, immediate hospitalization, and ongoing monitoring during recovery.^[5]^ Given the high volume of emergency cases and the complexity of cardiovascular diseases, triage nurses must make swift and accurate decisions to ensure timely care.

Optimizing emergency care processes and ensuring targeted interventions have proven effective in managing other conditions such as pancreatitis and cerebral hemorrhage.^[6–9]^ This study hypothesizes that, compared with traditional emergency care methods, optimizing emergency care processes can reduce treatment delays in AMI patients, improve clinical outcomes, enhance prognoses, and increase patient satisfaction. Therefore, the aim of this study is to evaluate the impact of optimized emergency care processes on the rescue and treatment outcomes of patients with AMI, as well as on the anxiety, depression, patient satisfaction, and complications experienced. In addition, the study will analyze the relationship between reduced rescue times and cardiac enzyme levels.

## 2. Method

### 2.1. General information

This study was approved by the Ethics Committee of Wuhan Third Hospital. A total of 201 patients diagnosed with AMI were admitted to our hospital’s emergency department from June 2022 to February 2024. Based on differences in prior emergency care processes, the patients were divided into an observation group (n = 93) and a control group (n = 108).

#### 2.1.1. Inclusion criteria

First onset of AMI; patients meeting the diagnostic criteria for AMI, characterized by ST-segment elevation or Q-wave elevation, presenting with persistent chest pain, a crushing sensation behind the sternum, and pain lasting for no <30 minutes, indicating typical myocardial ischemia; patients received by ambulance; treatment and nursing processes consistent with routine care or optimized emergency nursing; comprehensive historical information.

#### 2.1.2. Exclusion criteria

Presence of malignant tumors; other cardiac diseases; systemic autoimmune diseases; diseases of vital organs such as liver and kidney; cognitive impairment; recent surgery.

### 2.2. Nursing process

Both the observation and control groups adhered to the hospital’s standard antiplatelet therapy protocols to ensure uniformity in treatment. In the observation group, optimized emergency nursing protocols included the immediate initiation of dual antiplatelet therapy with aspirin and a P2Y12 inhibitor upon diagnosis, coordinated with the rapid administration of these medications during prehospital and in-hospital phases. The timing of antiplatelet therapy initiation was meticulously recorded, ensuring that patients began their treatment within 15 minutes of hospital admission. In contrast, the control group received standard care, where dual antiplatelet therapy was initiated upon arrival at the emergency department, typically within 30 minutes of admission. This difference in timing was part of the optimized process aimed at reducing rescue times and improving clinical outcomes.

The emergency nursing process for the control group primarily included comprehensive assessment of the patient’s condition, provision of routine emergency nursing care, timely PCI treatment, and strict monitoring after treatment. Nursing measures covered oxygen administration, blood sampling, electrocardiogram monitoring, and blood pressure monitoring, ensuring comprehensive examination and treatment for patients after admission.

Observation group (optimized emergency nursing process): prehospital emergency care: receiving alarms and departure: upon receiving a 120 emergency call, the emergency department quickly assesses the patient’s condition and prepares emergency supplies within 5 minutes, then departs for the call. Medical staff provide telephone guidance to the family for basic first aid (e.g., psychological comfort, bed rest). On-site assessment and rescue: upon arrival, the patient’s vital signs are immediately assessed, and airway patency is ensured. A rapid intravenous line is established, and the patient is moved to the ambulance with gentle handling to avoid discomfort. Transport monitoring: continuous observation of the patient’s condition is performed in the ambulance, with a mask for oxygenation to ensure patient comfort. The hospital is notified 10 minutes in advance to activate the green channel. In-hospital emergency care: emergency room reception: upon arrival at the emergency room, oxygen is immediately administered, and vital signs are monitored. Blood samples are collected for myocardial enzyme tests and electrocardiogram checks. Rapid decision-making and surgical preparation: after confirming AMI, arrangements for PCI surgery are made immediately. Nursing staff communicate with the family to prepare for the procedure (comfort, psychological support, medication). Standardized nursing process: a detailed nursing process chart is established to document each stage from emergency care to treatment. The responsible nurse’s duties must be clear, including preliminary electrocardiogram identification and assessment. postoperative care: monitoring and guidance: postsurgery, the patient’s vital signs and condition changes are closely monitored. Guidance is provided to patients and families regarding medication adherence and dietary recommendations. Psychological support and education: nursing staff should enhance psychological counseling for patients, maintaining emotional stability. Education on myocardial infarction should be provided to patients and families to improve compliance. Optimized management and communication: implementation of the first-responsibility system: nursing staff take turns on duty, allocating patients reasonably to ensure no shirking of responsibility. Communication mechanism: a handover process for patients with AMI is established to ensure 24-hour physician availability and timely response. Environmental optimization: Convenience measures are set up in the emergency hall to improve the consultation environment and enhance patient comfort. Through these optimized processes, the efficiency and success rate of treating patients with acute myocardial infarction can be effectively improved, while also focusing on psychological support to promote comprehensive recovery.^[8]^

Nursing interventions for both groups ended once the patient’s condition stabilized and they were transferred to a general ward, followed by an evaluation of the nursing outcomes.

### 2.3. Data collection

1. General information on the onset: age, gender, time from onset to admission, Killip classification, classification of myocardial infarction location, history of coronary heart disease, and relevant medical history.
2. Emergency information: time of emergency response, triage assessment time, time for emergency electrocardiogram (ECG) testing, and time to establish an intravenous line.^[9]^
3. Examination information: cardiac enzyme profile and electrocardiogram.
4. Self-Rating Anxiety Scale (SAS): the SAS is used to assess individual anxiety levels, consisting of 20 items scored on a 4-point scale. Higher scores indicate higher levels of anxiety.
5. Self-Rating Depression Scale (SDS): the SDS is used to assess individual depressive symptoms, also containing 20 items, scored on a 4-point scale. Higher scores indicate more severe depression.
6. Hert Hope Index (HHI): the HHI is used to assess individual hope levels, including 12 items scored on a 4-point scale. Higher scores indicate stronger feelings of hope.
7. Nursing satisfaction: collected using a questionnaire.

### 2.4. Propensity score

Propensity score is a method used to reduce confounding bias in observational studies, especially in nonrandomized situations. By conducting regression analysis on patient background factors, the propensity score estimates the probability of each individual receiving a specific treatment. This score can then be used for matching, stratification, or weighting analyses to achieve better balance between the treatment and control groups, thereby reducing the impact of confounding factors on the outcomes. In this study, matching was performed based on patients’ age, gender, time from onset to admission, Killip classification, classification of myocardial infarction location, history of coronary heart disease, and relevant medical history information.

### 2.5. Statistical analysis

Statistical analysis for this study was performed using SPSS software (SPSS Statistics 25.0, IBM, Armonk). For continuous variables, the Shapiro–Wilk test was conducted to determine if the data followed a normal distribution. If the data were normally distributed, mean ± standard deviations were used for description, and independent samples *t* test was employed for intergroup comparisons. If the data did not follow a normal distribution, medians and interquartile ranges were used for description, and 1-way ANOVA was conducted to compare group differences. Categorical variables were presented as frequencies or percentages, with differences between groups assessed using chi-square tests. A *P* value of <0.05 was considered statistically significant, indicating that the observed differences were not due to random error.

## 3. Results

### 3.1. General information

**Figure 1.**
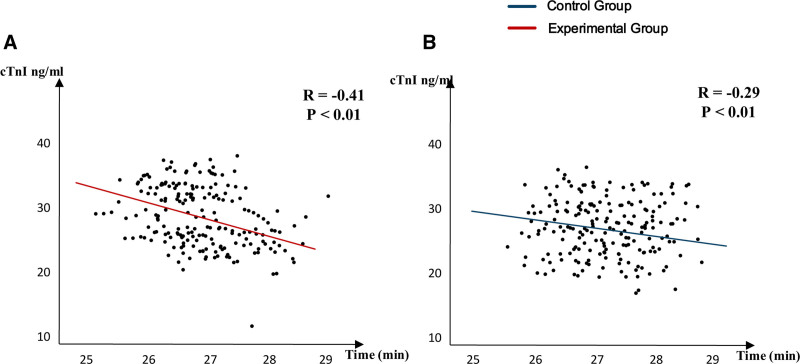
Correlation analysis between myocardial enzyme fall and emergency time.

At the beginning of the study, we statistically analyzed the basic information regarding age, gender, time from onset to admission, Killip classification, classification of myocardial infarction location, history of coronary heart disease, and relevant medical history between the 2 groups, as shown in Figure 1. The table on the left indicates that there were significant differences between the 2 groups in terms of age, time from onset to admission, Killip classification, and history of coronary heart disease (*P* < .05). Therefore, to eliminate the influence of confounding factors between the groups, we performed matching on the overall basic information, providing a solid statistical foundation for subsequent comparisons. After matching, as shown in the right half of Table 1, there were no statistical differences in basic information between the 2 groups (*P* > .05).

**Table 1:** Basic information.

Variables	Before matching			After matching		
	Experimental group (n = 93)	Control group (n = 108)	*P*	Experimental group (n = 85)	Control group (n = 85)	*P*
Age, yr	53.8 ± 1.2	49.8 ± 1.7	.043	52.1 ± 1.4	51.9 ± 1.5	.745
Gender			.054			.537
Male	54	48		49	45	
Female	39	60		36	40	
Time from onset to admission, h	6.45 ± 2.37	6.17 ± 2.14	.039	6.32 ± 2.48	6.29 ± 2.59	.066
Killip classification			.005			.664
Killip I	11	33		11	16	
Killip II	41	31		35	30	
Killip III	32	29		30	28	
Killip IV	9	15		9	11	
Myocardial infarction location			.767			.916
Anterior myocardial interwall type	41	53		37	37	
Inferior myocardium	33	34		32	30	
Anterior myocardial wall	19	21		17	19	
History of coronary heart disease, yr	8.46 ± 2.51	9.32 ± 4.65	.044	8.97 ± 4.51	9.06 ± 4.41	.077
Previous history						
Hypertension	26	30	.978	20	23	.987
Diabetes	35	41	.954	35	36	.998
Propensity score ($\bar{X}\pm S$)	0.23 ± 0.13		<.01	0.04 ± 0.06		.110

### 3.2. Comparison of the optimized nursing process on rescue and treatment outcomes

First, we compared the rescue indicators and treatment outcomes within the entire process. The rescue indicators included rescue time, triage assessment time, ECG testing time, and the time taken to establish an intravenous line. These indicators reflect the emergency capabilities of the process and the reduction of emergency response times due to the intermediate connections, as shown in Table 2. Compared with the control group, the experimental group had significantly shorter times for all indicators, with rescue times of 39.64 minutes for the experimental group and 46.98 minutes for the control group.

**Table 2:** Information comparison between rescue index and rescue effect.

Group	Rescue effect				Therapeutic effect		
	Rescue time (min)	Triage assessment time (min)	Emergency ECG test time (min)	Establish infusion channel time (min)	ST retreat (%)	Chest pain relief (%)	Myocardial enzyme pattern fell back (%)
Experimental group (n = 85)	39.64 ± 1.91	4.51 ± 0.82	3.45 ± 2.61	4.87 ± 1.36	50.59	62.35	70.59
Control group (n = 85)	46.98 ± 2.10	6.30 ± 0.61	5.81 ± 1.87	5.41 ± 1.08	29.41	48.24	49.41
T/X^2^ value	11.36	2.11	2.41	1.39	0.759	0.315	0.844
*P* value	<.01	.037	.011	.029	.034	.043	.017

ECG = electrocardiogram.

Subsequently, we evaluated and compared the treatment outcome indicators for both groups, which included ST-segment resolution, relief of chest pain, and the decline in cardiac enzyme levels. The results showed that the occurrence of treatment indicators in the experimental group was significantly higher than that in the control group. Specifically, the resolution of cardiac enzyme levels occurred in 70.59% of the experimental group, while it was 49.41% in the control group.

### 3.3. Relationship between optimized emergency nursing process, reduced rescue time, and cardiac enzymes

Previous studies have shown that the golden emergency time for patients with myocardial infarction is significantly related to changes in cardiac injury. Therefore, based on the aforementioned findings, we conducted a correlation analysis between the decline in cardiac enzyme levels and rescue time for both the experimental and control groups, selecting typical cardiac enzymes for myocardial infarction, such as Troponin I, as shown in Figure 1. It is evident that there is a significant negative correlation between cardiac enzyme levels and rescue time in both groups, with the experimental group showing a negative correlation coefficient of −0.41, while the control group exhibited a negative correlation coefficient of −0.29.

### 3.4. Impact of the optimized emergency nursing process on patients’ anxiety and depression

In addition, based on the characteristics of the optimized emergency nursing process, we aimed to alleviate patients’ emotional distress in addition to treating their medical conditions. Therefore, we compared the psychological scores related to patients between the 2 groups, including the SAS, SDS, and HHI, as shown in Table 3. Before receiving care, there were no significant differences in the 3 psychological scores between the 2 groups. However, after receiving care, the SAS and SDS scores in the experimental group were significantly lower than those in the control group, while the HHI score was significantly higher than that in the control group. Specifically, the SAS scores for the 2 groups were 47.45 and 53.11, respectively, the SDS scores were 51.11 and 58.72, respectively, and the HHI scores were 28.77 and 27.01, respectively.

**Table 3:** Comparison of anxiety and depression scores.

Group	SAS		SDS		HHI	
	Precare	After care	Precare	After care	Precare	After care
Experimental group (n = 85)	62.16 ± 6.33	47.45 ± 4.59	63.71 ± 4.12	51.11 ± 4.33	25.76 ± 2.03	28.77 ± 2.10
Control group (n = 85)	61.99 ± 6.43	53.11 ± 5.13	63.41 ± 3.97	58.72 ± 4.09	25.53 ± 2.32	27.01 ± 1.98
*T* value	0.841	5.413	0.613	7.151	0.411	1.451
*P* value	.656	<.001	.845	<.001	.774	.041

HHI = Hert Hope Index, SAS = Self-Rating Anxiety Scale, SDS = Self-Rating Depression Scale.

### 3.5. Impact of the optimized emergency nursing process on patients’ complications

We also conducted a statistical analysis of the occurrence of complications between the 2 groups, as shown in Table 4. There were significant differences between the 2 groups regarding the incidence of secondary PCI and AMI recurrence. Specifically, the proportion of secondary PCI in the experimental group was 11.76%, while it was 23.53% in the control group. The recurrence rates of AMI were 8.24% for the experimental group and 5.88% for the control group. No significant differences were found between the 2 groups regarding thrombosis, infection, or pressure ulcer occurrences.

**Table 4:** Comparison of complications between the 2 groups.

	Secondary PCI (%)	AMI recurrence	Thrombus	Infect	Pressure sore
Experimental group (n = 85)	11.76	8.24	0	2.35	0
Control group (n = 85)	23.53	5.88	1.18	3.53	1.18
*X*^2^ value	0.574	0.873	0.043	0.112	0.132
*P* value	.012	.041	.757	.664	.441

AMI = acute myocardial infarction, PCI = acute myocardial infarction.

### 3.6. Comparison of nursing satisfaction between different nursing approaches

Finally, we compared the nursing satisfaction levels of patients in both groups. There were significant differences in satisfaction with basic nursing and emergency nursing; however, no significant differences were found regarding nursing professionalism. Specifically, the satisfaction levels for basic nursing were 93.16% in the experimental group and 85.75% in the control group, while the satisfaction levels for emergency nursing were 93.43% in the experimental group and 88.65% in the control group, as shown in Table 5.

**Table 5:** Comparison of nursing satisfaction.

	Primary care	Emergency care	Nursing specialty
Experimental group (n = 85)	93.16 ± 4.12	93.43 ± 3.12	92.15 ± 4.11
Control group (n = 85)	85.75 ± 3.76	88.65 ± 4.32	91.16 ± 5.21
*X*^2^ value	9.45	5.64	0.022
*P* value	<.001	<.001	.664

## 4. Discussion

AMI has a rapid onset and high mortality rate, making timely rescue crucial. The emergency treatment of AMI must seize the time window to perform PCI as early as possible to unblock occluded vessels, improve myocardial blood flow perfusion, and save critically endangered myocardial cells. The key to this rescue process lies in the management of the time window. Traditional emergency nursing processes often delay optimal rescue times due to cumbersome procedures, delayed information transmission, and unreasonable process planning, negatively impacting patient treatment outcomes. PCI has a significant therapeutic effect on acute myocardial infarction, with higher cure rates when reperfusion treatment is initiated earlier after the onset of symptoms. Research indicates that controlling the time from admission to balloon dilation within 90 minutes can significantly improve rescue success rates.^[10]^ Therefore, optimizing emergency nursing processes is particularly important. By rationally integrating hospital resources, clarifying nursing staff roles, implementing seamless handovers and rapid transportation, and combining timely psychological interventions during the rescue process, it is possible to effectively shorten rescue preparation times, reduce myocardial damage, and enhance treatment outcomes.^[11–13]^ The optimized nursing process not only improves rescue efficiency but also gains valuable time for timely PCI and other critical treatments, thereby significantly improving patient prognosis and reducing mortality rates. This study demonstrates that scientifically standardized optimization of emergency nursing is a key measure for enhancing the success rate of AMI rescues.

Firstly, the optimized nursing process improves the systematic, scientific, and effective nature of nursing work through clear division of labor and standardized operations. Specifically, optimizing prehospital rescue steps allows for the rapid completion of material preparation, remote guidance, comprehensive assessment, and targeted interventions, thereby stabilizing the patient’s condition and gaining precious time for emergency treatment upon arrival.^[14–16]^ In addition, optimizing transport nursing clarifies nursing goals and standardizes procedures, effectively shortening transport handling times and ensuring patient safety during transport. The optimization of in-hospital rescue further shortens the preoperative preparation time for PCI, enhancing overall rescue efficiency and effectiveness. Experimental data show that the experimental group had significantly shorter rescue times, triage assessment times, ECG testing times, and intravenous line establishment times, with significantly higher success rates for ST-segment resolution, relief of chest pain, and decline in cardiac enzyme levels compared to the control group (*P* < .05). Second, the optimized emergency nursing process not only focuses on patients’ physiological health but also emphasizes interventions for psychological well-being. Through comprehensive caregiving, patients’ treatment confidence is enhanced, helping them maintain a positive mindset, which further improves treatment outcomes. The study found that the SAS, SDS, and HHI scores for patients in the experimental group were significantly better than those in the control group (*P* < .05). Finally, this study also conducted a correlation analysis between cardiac enzyme decline and rescue time for both the experimental and control groups, revealing that the decline in cardiac enzyme levels was significantly negatively correlated with rescue time in both groups, indicating that shorter rescue times lead to faster recovery of cardiac enzymes. Therefore, optimizing emergency nursing processes can effectively shorten rescue times, alleviate myocardial damage, and significantly improve patient prognosis.^[17,18]^

Another study utilized the Strengths, Weaknesses, Opportunities, Threats analysis method to optimize the emergency process for patients with AMI and applied a medical care integration intervention model to nursing. The results showed that the incidence of symptomatic cerebral hemorrhage and mortality rates in the research group were significantly lower than in the control group (*P* < .05),^[9]^ demonstrating the effectiveness of this method in optimizing AMI emergency procedures, reducing complications and mortality rates, and improving prognosis. This result is consistent with our study, which also analyzed patients’ psychological health and the relationship between rescue time and cardiac enzymes.^[10]^ Zhang et al^[8]^ grouped patients and adopted a tiered nursing model, showing that waiting, triage, and resuscitation times in the experimental group were significantly shorter than those in the control group (*P* < .05). In addition, the accuracy of diagnoses and resuscitation success rates were higher, with fewer complications and significant improvements in Karnofsky Performance Status and quality of life scores, indicating the effectiveness of the tiered nursing model in emergency care. These studies underscore the importance of optimizing emergency nursing processes. Our research further refines the emergency process into 4 components, improving the systematic approach to rescuing patients with AMI.

This study does have some limitations. First, as this study is a retrospective analysis, there may be selection bias in choosing samples. Second, the sample size in this single-center study is relatively small. We plan to conduct a multicenter randomized controlled trial while developing and optimizing a more reasonable emergency nursing process to observe patients’ long-term recovery outcomes.

## 5. Conclusion

This study demonstrates that optimizing the emergency nursing process can significantly shorten the rescue time for patients with AMI, improve treatment outcomes, and enhance patient prognosis. The optimized nursing process not only reduces rescue delays but also improves patients’ psychological states, significantly increasing nursing satisfaction and the success rate of cardiac enzyme decline. Optimizing emergency nursing processes can be promoted in clinical practice.

## Author contributions

**Conceptualization:** Fang Yu, Xueqin Yuan, Shouzhi Fu.

**Data curation:** Fang Yu, Xueqin Yuan, Shouzhi Fu.

**Formal analysis:** Fang Yu, Xueqin Yuan, Shouzhi Fu.

**Investigation:** Fang Yu, Xueqin Yuan, Shouzhi Fu.

**Methodology:** Fang Yu, Xueqin Yuan, Shouzhi Fu.

**Writing – original draft:** Fang Yu, Xueqin Yuan.

**Writing – review & editing:** Fang Yu, Xueqin Yuan.

**Funding acquisition:** Shouzhi Fu.

**Validation:** Shouzhi Fu.

## References

[1] Jiang K Tu Z Chen K. Gasdermin D inhibition confers antineutrophil-mediated cardioprotection in acute myocardial infarction. J Clin Invest. 2022;132:e151268. 34752417 10.1172/JCI151268 PMC8718151

[2] Hsieh YK Wang MT Wang CY Chen CF Ko YL Huang WC. Recent advances in the diagnosis and management of acute myocardial infarction. J Chin Med Assoc. 2023;86:950–9. 37801590 10.1097/JCMA.0000000000001001 PMC12718803

[3] Dreyer RP Sciria C Spatz ES Safdar B D’Onofrio G Krumholz HM. Young women with acute myocardial infarction: current perspectives. Circ Cardiovasc Qual Outcomes. 2017;10:e003480. 28228455 10.1161/CIRCOUTCOMES.116.003480 PMC5502480

[4] Lee SH Kim HK Ahn JH. Prognostic impact of hypercoagulability and impaired fibrinolysis in acute myocardial infarction. Eur Heart J. 2023;44:1718–28. 36857519 10.1093/eurheartj/ehad088

[5] Schuur JD Baugh CW Hess EP Hilton JA Pines JM Asplin BR. Critical pathways for post-emergency outpatient diagnosis and treatment: tools to improve the value of emergency care. Acad Emerg Med. 2011;18:e52–63. 21676050 10.1111/j.1553-2712.2011.01096.x PMC3717297

[6] Clark EG Watson J Leemann A. Acute care needs in an Indian emergency department: a retrospective analysis. World J Emerg Med. 2016;7:191–5. 27547278 10.5847/wjem.j.1920-8642.2016.03.005 PMC4988108

[7] Noureddine S Dumit NY Saab M. Deciding to seek emergency care for acute myocardial infarction. Clin Nurs Res. 2015;24:487–503. 25165070 10.1177/1054773814548508

[8] Zhang Q Yu Y. Effects of graded emergency nursing on resuscitation outcomes, prognosis, and nursing satisfaction in patients with acute myocardial infarction. Am J Transl Res. 2021;13:10586–92. 34650730 PMC8507081

[9] Wu C Wu L Jin P. Effect of SWOT analysis combined with the medical and nursing integration emergency nursing process on emergency treatment efficiency and prognosis of patients with acute myocardial infarction. Emerg Med Int. 2022;2022:7106617. 35941962 10.1155/2022/7106617 PMC9356903

[10] Pendyal A Rothenberg C Scofi JE. National trends in emergency department care processes for acute myocardial infarction in the United States, 2005 to 2015. J Am Heart Assoc. 2020;9:e017208. 33047624 10.1161/JAHA.120.017208 PMC7763391

[11] Bjöhle S Vicente V Eriksson C. Prehospital emergency nurses’ experiences of caring for patients with suspected acute myocardial infarction: an interview study. BMJ Open. 2024;14:e088754. 10.1136/bmjopen-2024-088754 PMC11409269 39260870

[12] Andersson H Ullgren A Holmberg M Karlsson T Herlitz J Sundström BW. Acute coronary syndrome in relation to the occurrence of associated symptoms: a quantitative study in prehospital emergency care. Int Emerg Nurs. 2017;33:43–7. 28438478 10.1016/j.ienj.2016.12.001

[13] Wang L Hao X Zou W Zhang D Huo H. Optimizing the effects of a comprehensive emergency nursing model on rescued patients with acute myocardial infarction in the cardiology department [published online ahead of print June 21, 2024]. Altern Ther Health Med. 38904636

[14] Ward MJ Baker O Schuur JD. Association of emergency department length of stay and crowding for patients with ST-elevation myocardial infarction. West J Emerg Med. 2015;16:1067–72. 26759656 10.5811/westjem.2015.8.27908 PMC4703176

[15] Bettencourt N Mateus P Dias C. Impact of pre-hospital emergency in the management and prognosis of acute myocardial infarction. Rev Port Cardiol. 2005;24:863–72. 16121677

[16] Li PWC Yu DSF Yan BP Wong CW Yue SCS Chan CMC. Effects of a narrative-based psychoeducational intervention to prepare patients for responding to acute myocardial infarction: a randomized clinical trial. JAMA Netw Open. 2022;5:e2239208. 36306128 10.1001/jamanetworkopen.2022.39208 PMC9617174

[17] de Cordova PB Johansen ML Martinez ME Cimiotti JP. Emergency department weekend presentation and mortality in patients with acute myocardial infarction. Nurs Res. 2017;66:20–7. 27977565 10.1097/NNR.0000000000000196

[18] Sanders SF DeVon HA. Accuracy in ED triage for symptoms of acute myocardial infarction. J Emerg Nurs. 2016;42:331–7. 26953510 10.1016/j.jen.2015.12.011

